# Community-Engaged Intervention Mapping for Cardiovascular Disease Prevention in Black and Latinx Sexual Minority Men With HIV in New York City: Protocol for a Web-Based Mixed Methods Study

**DOI:** 10.2196/41602

**Published:** 2022-10-11

**Authors:** S Raquel Ramos, Marilyn Fraser, Faven Araya, Hyun Young Kim, Jon Andre Sabio Parrilla, Kalla Maxine Sy, Riya Tongson Nagpal, Marlene Camacho-Rivera, Mohamed Boutjdir

**Affiliations:** 1 School of Nursing Yale University Orange, CT United States; 2 School of Public Health, Social and Behavioral Sciences Yale University New Haven, CT United States; 3 Arthur Ashe Institute for Urban Health Brooklyn, NY United States; 4 College of Nursing New York University New York, NY United States; 5 School of Public Health State University of New York Downstate Health Sciences University Brooklyn, NY United States; 6 Cardiovascular Research Program VA New York Harbor Healthcare System Brooklyn, NY United States; 7 Department of Medicine, Cell Biology and Pharmacology State University of New York Downstate Medical Center Brooklyn, NY United States; 8 Department of Medicine New York University School of Medicine New York, NY United States

**Keywords:** intervention mapping, CVD, eHealth intervention, HIV, sexual minority men, Black men, Latinx, community engagement, men who have sex with men, community based, qualitative, survey

## Abstract

**Background:**

Approximately every 37 seconds, someone in the United States dies of cardiovascular disease (CVD). It has emerged as an important contributor to morbidity among persons with HIV. Black and Latinx sexual minority men are at higher risk of both HIV and CVD when compared to heterosexual, nonethnic or minority men. Persons with HIV have a 1.5 to 2-times risk of having CVD than do HIV-negative persons. Data suggest that by the year 2030, an estimated 78% of persons with HIV will have CVD. The relationship between HIV and CVD in marginalized populations is not well understood because overall awareness of HIV and CVD as comorbid conditions is low, which further heightens risk. This has created a critically pressing issue affecting underrepresented ethnic and racial populations with HIV and requires immediate efforts to mitigate risk.

**Objective:**

The purpose of this formative, mixed methods study is to use a community-engaged approach to map a behavioral intervention for CVD prevention in Black and Latinx sexual minority men with HIV in New York City.

**Methods:**

Literature reviews focused on behavioral prevention studies using intervention mapping. In Aim 1, we will use qualitative interviews with HIV program managers and community members to understand facilitators and barriers to CVD prevention, chronic illnesses of concern, and early design elements needed for a web-based CVD prevention intervention. In Aim 2, we will conduct qualitative interviews and administer cross-sectional validated surveys with 30 Black and Latinx sexual minority men with HIV. We will assess illness perceptions of chronic conditions, such as HIV, hypertension, and diabetes. A total of 40 participants (program managers and community members) for Aims 1 and 2 will be enrolled to participate. To develop the protocol, we will follow steps 1 through 3 (needs assessment, change objectives, implementation strategy) of intervention mapping, using mixed methods.

**Results:**

The study was approved by New York University Institutional Review Board in February 2021 (IRB-FY2021-4772) and also by the Yale University Institutional Review Board in June 2022 (#2000031577). We anticipate completing data collection on or before December 2022. Early analyses suggested concerns about illnesses outside of HIV and associated comorbid conditions, such as COVID-19 and monkeypox. Additionally, we noted a strong interest in using a web-based platform for CVD prevention education.

**Conclusions:**

Web-based, behavioral, CVD prevention interventions may be promising modalities to closing the cardiovascular health disparities gap in Black and Latinx sexual minority men with HIV by extending the reach of prevention interventions using community-informed approaches and technological modalities that have been underused in this population.

**International Registered Report Identifier (IRRID):**

RR1-10.2196/41602

## Introduction

### Background

Cardiovascular disease (CVD) and HIV are 2 chronic conditions that are often comorbid in the presence of HIV. Persons with HIV are at higher risk of CVD than are HIV-negative persons. Specifically, persons with HIV have a 1.5 to 2-times higher risk of CVD [1]. In the United States, a person dies of CVD approximately every 37 seconds [2]. According to 2018 data, it is the leading cause of death in the United States and accounts for more deaths than does cancer and chronic respiratory illnesses combined [2]. In 2019, it was estimated that over 18 million deaths were due to CVD [2]. The consequences of CVD are also costly, with average expenditures in 2016-2017 estimating upward of US $364 billion dollars [2]. Although the costs of CVD are high, these costs exponentially increase when combined with other chronic conditions, such as HIV.

Higher CVD risk in those with HIV is linked to increased inflammation and hyperstimulation of the immune system [3]. The analysis of 3 large international HIV treatment trials suggested that higher interleukin 6 and D-dimer levels reflecting inflammation and coagulation associated with HIV are also associated with an increased risk of fatal CVD and a greater risk of death following a nonfatal CVD event [3]. Recent publications linked CVD with inflammation and antiretroviral treatment associated with HIV [4,5]. Furthermore, risk factors of coronary heart disease (eg, smoking, diet) as well as nontraditional risk factors (eg, hepatitis C, substance use) should also be considered in persons with HIV [5,6]. A 2020 study noted the existence of promising treatments and medications for inflammation and atherosclerotic CVD (ASCVD); however, these treatments have yet to show similar significant results for treating HIV-associated CVD [5]. Estimated 10-year CVD risk is highest in Black men (8.3%) and Latinx men (4.4%) with HIV when compared to nonminoritized HIV-negative individuals [7] and remains a significant pressing health issue toward cardiovascular health equity.

### HIV Health Disparities Impacting Advancement of Cardiovascular Health in Sexual Minority Men

Over the course of 9 years (2010-2019), the incidence in HIV diagnoses has shifted disproportionally in racial and ethnic populations [8]. For example, in 2021, sexual minority men (herein referring to persons who identify as nonheterosexual, gay, bisexual, queer, or same-gender loving) accounted for two-thirds of all new HIV infections [8]. Black and Latinx populations have shown little to no decrease in HIV diagnoses [8].Whereas, the number of new HIV infections in White persons decreased from 7500 to 5100 (per 100,000 people) [8]. Data trends suggest that HIV prevention efforts have fared better in nonminoritized ethnic and racial populations. By the year 2030, an estimated 78% of persons with HIV will have CVD [9]. A 2016 study examining CVD risk in a West African population showed that for every 10 participants in their 256 person sample, 8 had at least 2 CVD risk factors [10]. The prevalence of CVD in persons of color is significant and calls for immediate interventions [11], especially within sexual minority men of color with HIV. To address this, interventions must be focused on improving cardiovascular health and be tailored to communities who are at highest risk.

### Matrices of Cardiovascular Risk

The American Heart Association created the Life’s Simple 7 (LS7), a tool developed to assess cardiovascular health risks. The overall goal of this tool is to promote cardiovascular health related to 4 modifiable lifestyle risk factors (diet, smoking, BMI, physical activity) and 3 biometric measures (blood pressure, cholesterol, and blood sugar) [11]. Each category is divided into 3 scoring levels (poor = 0, intermediate =1, ideal =2) with an optimal total score of 11 to 14, an average score of 9 to 10, and an inadequate score of 0 to 8. By using this tool, areas for improvement in cardiovascular health can be identified in those with HIV who carry high CVD risk.

A longitudinal study comparing the different risk factors of ASCVD within persons with HIV observed that age, diabetes mellitus, current smoking status, hypertension, and dyslipidemia were associated with an elevated risk of ASCVD [12]. Another study showed the use of LS7 lowered CVD risks by roughly 78% compared to those that did not follow any of the metrics [13]. The Reprieve study, a prospective, double-blind, placebo-controlled, multicenter, phase III efficacy trial, observed CVD risk factors in a sample of over 7500 persons with HIV. Findings suggested that ideal cardiovascular health was driven by lower BMI and less smoking [12]. HIV-specific risk prediction models have also been in use to study persons with HIV. The Data Collection on Adverse Events of Anti-HIV Drugs (D:A:D) model, developed in 2010, provides additional CVD risk factors that include CD4 counts, use of abacavir, and cumulative use of nucleoside reverse transcriptase inhibitors and protease inhibitors [14,15]. Although the D:A:D study incorporated a persons-with-HIV cohort that was predominantly European [7], it is important to note the additional factors that should be considered in this population, specifically assessing diverse populations, genders, and populations in low-and middle-income countries with HIV [16]. LS7 and the D:A:D model are promising tools to facilitate improvements in cardiovascular health and CVD risk in persons with HIV [16,17].

### Formative Development of a Community-Engaged, Web-Based CVD Prevention Protocol for Persons With HIV

To address the prevention of CVD and improve the cardiovascular health equity in Black and Latinx sexual minority men who are at the greatest risk, this study will use a tailored, community-engaged approach. Specifically, the study will be formative and intended to develop a protocol for a web-based, CVD behavioral intervention. The premise for this study originates from qualitative data from a larger parent study where preliminary findings suggest that sexual minority men of color with HIV are interested in technology-based, health-related, prevention education [18,19]. Additionally, there are limited studies using nonpharmacologic behavioral interventions to prevent CVD in sexual minority men of color with HIV [20]. To accomplish this goal, the study will be guided by intervention mapping. Intervention mapping prioritizes active participation of relevant stakeholders in the development process and acknowledges the complex intersectionality of influences (eg, individual, interpersonal, organizational, societal) on health outcomes [21]. Additionally, intervention mapping has been used in HIV research to foster collaboration between the researchers and the participants, allowing researchers to develop interventions that appeal to the needs of those they aimed to help [22]. These factors are key for the intervention program currently being developed, as levels of collaboration and common understanding between researchers and participants will help ensure that the partner communities are actively engaged in the intervention process. Intervention mapping consists of 6 steps but is by no means a linear process. The process is iterative and bidirectional with program developers moving between steps as they gain new information and perspective [23].

### Study Objectives

Acknowledging the lack of interventions to promote cardiovascular health equity in sexual minority men of color with HIV, we propose a tailored, community-engaged, web-based approach to prevention. Over the years, there has been an increase in web-based behavioral interventions. Given the ubiquity of technology, even among very low-income communities, web-based interventions have shown great promise as a modality for behavioral interventions. Using intervention mapping, participants will inform the researchers of intervention components that are necessary for engagement and knowledge building using a web-based platform. We have two aims: (1) engage 10 HIV program managers already engaged in health-related programming in New York City to explore the facilitators and barriers to developing a web-based, intervention mapping protocol for CVD prevention; and (2) develop a community-engaged, intervention mapping protocol for CVD prevention with 30 Black and Latinx sexual minority men. We anticipate that this formative work will result in a relevant and actionable protocol to be implemented using a web-based platform. The study design and objectives are advantageous to addressing the critical health equity gap in CVD incidence. They are also advantageous to increasing engagement and potential for behavioral change, as the protocol is informed by community members and not exclusively by empirical work. We expect that this will make the web-based content more relevant to the needs of participants, which in turn may increase recruitment, retention, and behavioral outcomes.

### Framework

Intervention mapping is an approach in which researchers design interventions through exploring the needs of the community [21]. Intervention mapping uses six steps: (1) develop a deeper understanding of the population and problem being studied, (2) determine the main issues of the overall problem and identify the best possible outcome if changes were made, (3) create intervention methods based on the identified issues in order to reach the positive outcomes, (4) use the intervention methods to create an intervention program, (5) implement the intervention program in the studied community, and (6) analyze the results of the program in order to determine the efficacy of the interventions [23].

Intervention mapping has been used to develop interventions and methods of care for a variety of diseases throughout different populations, ranging from HIV in young adult men [24] to diabetes in African American adults [25]. Researchers often combine the evidence learned from intervention mapping with other conceptual models, such as social cognitive theory or the transtheoretical model, to determine new avenues of preventative care [26]. In certain cases, the process of intervention mapping has been used as a reference to create new tools for more specific intervention development [26]. This study will incorporate formative steps 1 through 3.

### Steps to Intervention Mapping

#### Step 1: Logical Model of the Problem (Needs Assessment)

This step focuses on discussing the foundations of the issue being addressed to begin identifying possible changes for which interventions can be made. In this study, we focused on developing a community-engaged protocol to prevent CVD in Black and Latinx sexual minority men with HIV.

#### Step 2: Program Outcomes and Objectives—Logic Model of Change (Change Objectives)

This step focuses on needed changes and persons who are involved in improving the chosen health-related issue, which is CVD prevention. At this point, expected program outcomes are established through the identification of specified behaviors [27,28] required for increasing cardiovascular health. Environmental and behavioral outcomes are then differentiated into program objectives, and changeable determinants of behavior are selected for each program objective. For each expected program outcome, program objectives are aligned with the changeable determinants in a matrix design to identify the needed changes, or the change objectives. In this study, the researchers will construct a matrix based on prior literature reviews and the data collected during both study aims with HIV program managers and community members.

#### Step 3: Intervention Development (Framework Strategy)

A fundamental premise of the intervention mapping process is that all developed intervention methods are grounded in theory [28]. Having specified the objectives of the intervention based on the needs assessment in step 1 and the developed matrices of change in step 2, a relevant theoretical framework will be selected as the foundation of the intervention methods and strategies to create effective matrices of change.

### Diffusion of Innovations

Given the study’s focus on adoption of new ideas and behaviors, diffusion of innovations is an appropriate theory and will serve as a guide for this study. Diffusion of innovations [27] is an iterative process theory that focuses on understanding how “new ideas, practices and technologies” are spread through social networks and grow in familiarity to best facilitate adopting the innovation [29,30]. The theory proposes that there are five key [27] components to the successful uptake of innovative behavior: (1) the attributes of the innovation; (2) the adopters and their degree of innovativeness (ie, earliness to adoption); (3) the structure of the target social system and its opinion leaders, who can influence others’ attitudes or behaviors with relative frequency; (4) the individual adoption process; and (5) the diffusion system, comprising the change agency and its agents who introduce the desired innovation to the social system [29]. A change agency is a conglomerate of individuals (ie, change agents) that act as influencers for acceptance of the proposed innovation [27,31]. The influencers are often external to the targeted social network in which they are encouraging uptake of the proposed innovation [32,33].

Five categories of adopters exist in this theory: (1) innovators, (2) early acceptors, (3) early majority, (4) late majority, and (5) laggards [27]. Innovators, who often have high tolerance for ambiguity and often take more risk, are the very first people to adopt an innovation [27]. Early acceptors are next to adopt an innovation after judicious appraisal of an innovation’s advantages and disadvantages [27]. The early majority subsequently adopt an innovation due to social pressure, exhibiting an imitative effect [27]. The late majority are similarly influenced through social pressure but tend to be more skeptical and cautious [27]. Laggards, who are less susceptible to social pressure, take their time before adopting an innovation and may even resist the proposed innovation [27].

The theory has previously been applied across various innovative health interventions, such as an online-offline hybrid sexual health intervention among high-risk youth [34], a culturally specific individual-level peer navigation intervention among sexual minority men of color [35], and a peer-led social media–based intervention of HIV pre-exposure prophylaxis adoption [36]. For this study, the 5 key components of the diffusion of innovations theory are best suited to describe stages of adoption toward cardiovascular health.

## Methods

### Ethics Approval

All procedures performed in this study are in accordance with the ethical standards of the institutional and national research committee, the 1964 Helsinki Declaration and its later amendments, or comparable ethical standards. This study was funded by the National Heart, Lung, and Blood Institute (#R25HL105446) as a subaward through the SUNY Downstate Medical Center and through the Fund for Gay and Lesbian Studies (FLAGS), LGBT Studies at Yale University. Approval was obtained from the New York University Institutional Review Board in February 2021 (IRB-FY2021-4772) and by the Yale University Institutional Review Board in June 2022 (#2000031577). Informed consent will be obtained from all participants who meet the eligibility criteria and agree to participate. All participants who complete either Aim 1 or 2 will receive a US $45 gift card.

### Study Design

We are conducting a 2-phase, mixed methods, community-informed study in New York City.

Eligibility criteria includes the following: identifying as a sexual minority male (nonheterosexual), living with HIV, being a community member engaged in HIV programming in New York City, being 30 to 65 years old, having internet access, and self-identifying as being from a racial or ethnic minoritized background. It is documented that chronic illness is becoming diagnosed at earlier stages of life, with the highest prevalence at ages 50 years and above [37]. Data from the Million Hearts study found that more than 30% of life-changing cardiac events were in adults as young as 35 years of age [38]. Our sample age range is appropriate given the changing age demographic of chronic illness.

### Participant Recruitment

We are partnering with a premier urban health institute in New York City whose mission is to directly address health disparities through community-engagement and partnership to facilitate behavior change and informed health decision-making. Participants will be recruited from community-based organizations within New York City. Program managers will inform clients about the study through word of mouth, during activities with clients, and through posted or digital flyers. Interested persons who meet eligibility criteria will be scheduled to be consented and participate in the qualitative interviews. We anticipate that we will be able to successfully recruit a diverse sample by partnering with a community-based organization and because of the diversity of the New York City demographics. New York City (inclusive of its boroughs) is one of the most ethnic and racially diverse cities in the nation. According to the 2021 Census, New York City is 28.9% Hispanic and 23.8% Black [39]. The Bronx borough is 56.4% Hispanic and 43.8% Black, the Brooklyn borough is 18.8% Hispanic and 33.3% Black, and the Queens borough is 28.1% Hispanic and 20.7% Black [39]. Moreover, Behavioral Risk Factor Surveillance System (BRFSS) data suggest that 9.2% of adults in New York City self-identify as lesbian, gay, bisexual, or other [40].

### Procedures

#### Aim 1

In Aim 1, web-based focus group interviews using Zoom (Zoom Video Communications) with 10 key informants, including HIV program managers and community members at both the organizational and community levels, will be used to explore community-level barriers and facilitators to CVD prevention. Program managers are able to provide expert, public health– and community-informed perspectives about risk and external factors affecting prevention efforts. They can also address tailored strategies that may be helpful to mitigate CVD risk. It is important to include program managers and community members in Aim 1 to ensure both perspectives coalesce to provide a thorough and unbiased understanding of risk and community needs. A qualitative interview guide with 5 open-ended questions will be used, for example, “Tell me about how health-related community needs have been addressed in the past? How have strategies been most successful?” After completion of Aim 1, we will have identified barriers and facilitators (needs assessment) to community-based CVD prevention. These data will be used in the preliminary design (program objectives) of the intervention mapping protocol. Previous qualitative research approaches suggest that data saturation is reached when there is no longer new emerging data.

#### Aim 2

In Aim 2, web-based semistructured interviews will be conducted using Zoom with up to 30 community members, who will be individually interviewed, and will explore HIV-related chronic conditions of concern, barriers, and facilitators to CVD prevention. Qualitative interviews will explore technology-enabled design strategies and intervention components and materials that are responsive to identified community needs. After qualitative data collection for Aim 2, we will expect to have collected the necessary data (practical strategies, program components, and materials) for a tailored, behavioral, CVD prevention study. Additionally, to further examine health perceptions, engagement in physical activity, and nicotine use, we will administer survey questionnaires. Based on qualitative frameworks, our community members (N=30) is within standard sample size to achieve saturation [41,42] and to also provide sufficient descriptive data on HIV illness perceptions in order to make cautious inference using a small sample [43].

### Measures

#### International Physical Activity Questionnaire (Short Form)

The International Physical Activity Questionnaire (Short Form) is a 7-item questionnaire used in populations of adults between the ages of 15 and 64 years old. The questions are open-ended and require individuals to self-report their physical activity within the past 7 days. It has been used in several diverse populations internationally with young and middle-aged adults. An example question from this measure includes the following: “During the last 7 days, on how many days did you do vigorous physical activities like heavy lifting, digging, aerobics, or fast bicycling?” It has high reliability with a Cronbach α of <.80 and predictive validity [44].

#### Smoking Behaviors: The BRFSS

The BRFSS, is a 2-part telephone survey developed by the Centers for Disease Control and Prevention (CDC) to collect information regarding chronic conditions and health risk behaviors [45,46]. It is a survey used nationally as a tool to assess public health needs and priorities. We included questions on tobacco and e-cigarette use. Example questions included the following: “Have you smoked at least 100 cigarettes in your entire life?” “Have you ever used an e-cigarette or other electronic “vaping” product, even just one time, in your entire life?”

#### Illness Perception Questionnaire-Revised HIV

The Illness Perception Questionnaire-Revised (IPQ-R) for HIV has been adapted from the original IPQ by replacing illness with HIV [47]. The IPQ-R-HIV is an 83-item questionnaire that uses a Likert scale (1 = strongly disagree, 2 = disagree, 3 = neither agree nor disagree, 4 = agree, and 5 =strongly agree). The IPQ-R-HIV has a Cronbach α of .90 of and is reported to have acceptable internal consistency. The IPQ-R-HIV asks questions about views of living with HIV, symptoms associated with HIV, and symptoms associated with combination therapy. An example question from this measure includes the following: “Anti-HIV medication can control the progress of my HIV infection?” This measure has been previously tested in HIV studies with sexual minority men.

#### IPQ-R for Hypertension

The IPQ-R for Hypertension has been adapted from the original IPQ by replacing illness with hypertension [47]. The IPQ-R-Hypertension is an 80-item questionnaire that uses a Likert scale (1 = strongly disagree, 2 = disagree, 3 = neither agree nor disagree, 4 = agree, and 5 = strongly agree). The IPQ-R-Hypertension has a Cronbach α of 0.75 and is reported to have demonstrated good test‐retest reliability, and predictive, concurrent, and discriminant validity. The IPQ-R-Hypertension asks questions about hypertension representation, cause, and identity. An example question includes the following: “Having this high blood pressure makes me feel anxious?” This measure has been studied in populations at risk for atrial fibrillation and other related chronic conditions.

#### IPQ-R for Diabetes

The IPQ-R-Diabetes has been adapted from the original IPQ by replacing illness with diabetes [47]. The IPQ-R-Diabetes is a 64-item questionnaire that uses a Likert scale (1 = strongly disagree, 2 = disagree, 3 = neither agree nor disagree, 4 = agree, and 5 = strongly agree). The IPQ-R-Diabetes has a Cronbach α of >0.7 of and is reported to have sufficient internal consistency. The IPQ-R-Diabetes asks questions about views on living with diabetes and symptoms associated with diabetes. An example question from this measure includes the following: “I have experienced pain since my diabetes?” This measure has been used in international studies with populations of individuals diagnosed with type 2 diabetes.

### Data Analysis

In Aim 1 and Aim 2, focus groups and semistructured interviews will be analyzed using content analysis in NVivo version 12 (QSR International). Qualitative content analysis is consistent with a formative study, as it is an inductive process and is used to develop themes using either focus groups or interviews [48].

For aim 2 questionnaires, quantitative data will be analyzed using SPSS version 28 (IBM Corp). Descriptive statistics (frequencies, means, SDs) will be used to characterize participants reported illness perceptions, 7-day physical activity, and nicotine use. We will use bivariate statistics to examine relationships between participant characteristics and reported illness perceptions. Since this is formative and noninterventional work, power analyses are not warranted.

## Results

This study was approved by New York University Institutional Review Board in February 2021 (IRB-FY2021-4772) and by the Yale University Institutional Review Board in June 2022 (#2000031577). As of July 2022, we have completed data collection on over 90% of our anticipated sample and expect to compete all data collection on or before December 2022. We expect that the qualitative data will be robust and inform the necessary components for a community-informed and tailored intervention mapping protocol for CVD prevention in persons with HIV. The quantitative data will provide additional context into illness perceptions about HIV and also hypertension and diabetes, if applicable. Physical activity and nicotine use will provide further context on self-reported behavioral cardiovascular risk. Early qualitative analyses suggests concerns about conditions outside of HIV, hypertension, and diabetes, such as cancer, breathing problems, COVID-19, and monkeypox. We will have clearer insights once data collection has ended and data are analyzed on the complete sample.

## Discussion

### Expected Findings

The overarching aim of this formative study is to develop a community-engaged intervention mapping protocol for a web-based CVD prevention intervention in Black and Latinx sexual minority men with HIV ages 30 to 65 years. As Black and Latinx sexual minority men with HIV are at a higher relative risk for CVD, culturally competent and relevant interventions are necessary. There is a gap in the literature regarding culturally competent CVD preventative interventions for lesbian, gay, bisexual, transgender, queer, and others (LGBTQ+) populations, and this formative study addresses this cardiovascular health equity gap. We anticipate that the qualitative and quantitative data will inform us of the necessary information required for designing content for a CVD prevention, sexual minority–focused, web-based intervention in order to have maximal impact. Specifically, by using mixed methods, we will have a stronger understanding on how to tailor a CVD prevention intervention and also gain new knowledge about HIV, hypertension, diabetes, and other illness priorities in Black and Latinx men.

Technology-enabled interventions can be leveraged as innovative tools to mitigate chronic illness, as 62% of adults living with 1 or more chronic disease use online resources [49]. Technology-enabled, behavioral, CVD prevention interventions may be promising modalities to closing the cardiovascular health disparities gap in Black and Latinx sexual minority men with HIV. The Pew Research Center reported that the proportion of adults who have smartphones (and live in households with incomes less than US $30,000 thousand dollars per year) has increased by more than half [49]. Recent literature indicates that regardless of income level, a majority of persons have access to and are using the internet. According to the Williams Institute, 56% of Black LGBT adults live in low-income households (those which are below 200% of the United States federal poverty level) compared to 49% of Black heterosexual households [50]. Additionally, 37% of LGBT Latinx adults reside in a household with an annual income below US $24,000 per year [50]. This suggests increased uptake, accessibility, and use in smartphones and underscores the importance of using digital technologies to meet the increasing need of health education among Black and Latinx LGBT populations.

### Strengths and Limitations

The risk of CVD in Black and Latinx sexual minority men is a pressing health challenge. We address this challenge using a community-informed approach leveraging mixed methods data collection techniques and an established intervention mapping framework. Additionally, using a community-informed approach centers the perspectives of Black and Latinx sexual minority men with HIV, who have been historically been left-out of informing the design of CVD prevention interventions. We believe that using intervention mapping will result in the design of a tailored intervention strategy that will have relevance and increase engagement. By partnering with a community-based organization serving sexual minority men of color, we are able to develop an intervention strategy that focuses on the needs of the community and that is not based in the traditional clinical setting, as engagement may vary. We believe that these are strengths of the study. Moreover, the findings from this study will be disseminated back to the community through a verbal presentation and poster of the study findings. We also plan to disseminate study results in a high-impact journal and through scientific meeting presentations.

This study is not without limitations. First, generalizability is limited given the small sample size. However, although small, the sample size is within the recommended limits for qualitative and quantitative formative inquiry. Second, qualitative data collection using focus groups are subject to group dynamics which could influence external validity [51]. However, we are mitigating bias with having a trained moderator facilitate discussions. Third, self-reported measures are subject to social desirability and response bias. We are mitigating the potential for bias and missing data by having a trained investigator. Despite these limitations, the benefits of this study are an early, yet necessary step, in addressing a critical health disparity issue in persons with HIV.

### Conclusions

Formative, mixed methods approaches to developing web-based, community-engaged, behavioral interventions for CVD prevention in ethnic and racial sexual minority men with HIV hold much promise given the uptake of technology and internet use. Conventional approaches to CVD prevention, such as traditional patient teaching in the clinical setting, in overlooked, minoritized populations may not be feasible or sustainable given the differences in social determinants, culture, and overall health priorities.

## Abbreviations

ASCVD: atherosclerotic cardiovascular disease
BRFSS: Behavioral Risk Factor Surveillance System
CDC: Centers for Disease Control and Prevention
CVD: cardiovascular disease
D:A:D: Data collection on Adverse events of anti-HIV Drugs
FLAG: Fund for Gay and Lesbian Studies
IPQ-R: Illness Perception Questionnaire-Revised
LGBTQ+: Lesbian, gay, bisexual, transgender, queer, and others
LS7: Life's Simple 7

## Data Availability

The data that support the findings of this study will be available from the corresponding author (SRR) after data collection and dissemination activities have concluded. The data may be made available upon reasonable request.
